# Music and emotion—a composer's perspective

**DOI:** 10.3389/fnsys.2013.00082

**Published:** 2013-11-19

**Authors:** Joel Douek

**Affiliations:** One Track Music, Ltd., Private composerLos Angeles, CA, USA

**Keywords:** music, film, creativity, emotion, composers

## Abstract

This article takes an experiential and anecdotal look at the daily lives and work of film composers as creators of music. It endeavors to work backwards from what practitioners of the art and craft of music do instinctively or unconsciously, and try to shine a light on it as a conscious process. It examines the role of the film composer in his task to convey an often complex set of emotions, and communicate with an immediacy and universality that often sit outside of common language. Through the experiences of the author, as well as interviews with composer colleagues, this explores both concrete and abstract ways in which music can bring meaning and magic to words and images, and as an underscore to our daily lives.

It is a privilege to be invited to contribute to Frontiers in Neuroscience on this most fascinating and nefarious of topics. This article is not intended to be in any way scientific. Rather it is a more anecdotal look at the experiences of composing music, and in particular about driving human emotions through music to enhance moving images and dialogue. I attempt to work backwards from what we as practitioners of the art and craft of music do instinctively or unconsciously, and try to shine a light on it as a conscious process. Some of these thoughts will no doubt state the obvious, but I hope will also stimulate some discussion. Why should these things be in any way obvious? Because at heart there seems to be a core musical insight and expertise that pervades the human experience. There are universals in music that just do not exist in language. It is part of our humanity.

Almost every moment in a person's life is continually underscored by music—from our birth, to our wedding to our death, our celebrations, our wars and our victories. From casual listening at home, in a film or on TV, from an iPod as we move around or the sound of an ambulance racing by, a call to prayer, or just somebody at the door, music is there. Unlike eyes, ears can detect phenomena in any direction without having to be focused on it, giving distinct survival advantages throughout evolution (MPF, 2004). Vibration sense, sound sense, is ancient, visceral, and inextricably linked to old and deep emotional centers in the brain, a fact that allows composers to access and dialogue with their audience at a deep level.

Such is the influencing power of music, the brain states that it can induce that it is almost impossible not to get caught up. For the Sufis, music is a path to enlightenment, it is “food for the soul”^1^. In ancient shamanic traditions, it is a vehicle to travel into the deeper realms and heal a person (Harner, 1990). Jazz that open floodgate of intellectual freedom and abstraction, was famously outlawed by the Nazi Reichstag. Secular music finds itself frequently scorned by fundamentalist regimes, so much so that attitude toward music is a fairly accurate barometer of political and religious ideology. Yet despite the force of this influence, as Bob Marley said, “one good thing about music is when it hits you, you feel no pain” (lest the volume is too loud).

What makes music for film (and media in general) particularly interesting to any examination of how music affects emotions, is that in film that is its main function. As a veteran film composer once said: “In a film, the dialogue and action tell us what the characters are thinking and doing, but the music can tell us what they are feeling.” Music and movies have coexisted since film's beginning—there never really was a silent film. Before synchronized sound, there was always an accompanying instrument in the movie theater. If you watch a film without the music, it becomes impersonal, its energy and movement undetermined. Directors can even capitalize on the stark soullessness of a film without music. Devoid of music, the Coen Brothers' film “*No Country for Old Men”* (2007), has a coldness, an inhumanity that pierces right through you.

Writing music for film, unlike songs or concert music, is typically done within a multitude of confines and considerations. It must support a particular narrative and stay out of the way of dialogue, it may be required to draw from only a specific palette of instruments, and occur from only one point to another on the timeline. Unlike concert composers and songwriters, who can explore their creativity laterally, bound by these parameters film composers must explore vertically.

## How do you create music?

Beyond the tricks of the trade, I think most music writers are themselves mystified at where their ideas come from. Those that I asked “how do you create music?” answered simply “I have no idea.” The core of creating and articulating musical ideas seems to be 100% instinctive. Like an intuition or a reflex, it has an immediacy to it, and in that way it feels like an active interplay between unconscious and conscious experience. Something that you can never really control, but you can learn to harness.

And we try anything to coax out that pure spark that can flash for an instant and spawn a symphony, or a song. Perhaps it is this elusiveness that puts so many people in creative professions on the edge. Crisis and creativity are strange yet promiscuous bedfellows: perhaps it is because crisis demands a directness of experience. Maybe the raw emotions of duress clarify the path from emotion to musical expression. My own first real foray into composing was triggered by the torn emotions of a painful breakup. As one of my teachers puts it: “when you reach a place where you are truly out of options, where you don't care anymore, that's where you are free to begin.”

There is a double-edged sword to creative sensibility. To live in that paradoxical place where one can in an instant access the darkest of the dark to the lightest and most uplifting of emotions, leaves one determinedly exposed to “the slings and arrows of outrageous fortune.” It is a life lived intensely, and yet the experience is also intensely abstract.

So what are the skills that a composer needs? Are we psychologists or empaths? Probably yes, quite a bit of both. Does it require a heightened emotional maturity? Probably not. Mozart is a great example of an instinctual purity and brilliance that seemed to defy his lack of emotional maturity.

I think creativity embodies a kind of “constructive restlessness.” If not an attention deficit, then perhaps an attention brevity. The propensity for distraction, to go off at tangents, is a clear *advantage* to artists. Not to be stubborn, but to follow a whim, a creative hunch. Because it is at the edges of our awareness, where the tyrannical conscious mind is not really paying attention that the real magic happens. You catch an idea at the corner of your distraction, and you run with it.

While most people possess the capacity to feel emotions and have them triggered or enhanced by musical cues, composers need to have a kind of open conduit between feeling and fingers, a channel to move a complex, wordless inner experience toward an outward expression. We draw out these feelings from our well of experiences and influences, from what moves *us*. And we must take care to refill our well with new life adventures lest it run dry.

I see composing not as a process but a space. A convergence of creativity and craft into a perfect harmony we know as Inspiration, and it is best found within one's work, not without. Can it be learnt? While tricks and techniques can be learnt, I feel the ability to convey emotion through music is more innate, more personal. So if it is an instinctual thing, how does a film composer deal with having to deliver emotions made to order, and work on demand? By journeying into the emotional landscape of a scene, or into the head of the character in question (that's where the empathy or psychology comes in), lining up the elements that you can and then just letting the rest happen intuitively. If you set a place at the table, the Muse might come and join you.

Not all composers write the same way: some hear a symphony all at once, some, a poignant motif, a hook. Like many, I tend to improvise around ideas and begin to “hear what is not yet there, but should be.” A melody or harmony that is implied. I work with the information I have—chordal or modal structures that evoke the mood I need, a palette or rhythmic pace, and then the missing pieces become more conspicuous. The sensation of composing is often described as akin to “channeling,” perhaps from the unconscious to conscious mind, perhaps from somewhere more esoteric. Like a garden, you plant the seeds of ideas, water and nurture them, and if you have tended well, flowers and fruit will emerge.

## How do you create emotions with music?

One thing that film composers learn quickly is that when it comes to emotions, humans beings are much more music-driven than they are visual-driven. It takes only 25 frames per second to fool the eyes, maybe less. But you need thousands of samples per second to fool the ear. The immediacy with which we react to a film's soundtrack shows that at least emotionally, we are much more prejudiced to the sound than the images. One of my favorite examples of this bias is the recut trailer “*Scary Mary Poppins*” (link below)^2^, where the magically maternal Mary Poppins becomes an evil witch when underscored by creepy music.

In film editing, there is a well-known rule: “Sound Before Picture.” You will often hear sound from the next scene coming in before the visual images, and the brain is able to make perfect sense of it. We are propelled forward with a prescience of what follows. Not so when we bring visuals before their accompanying sound—that feels nonsensical and the brain is confused.

### Tips and tricks

Much of the work of the film composer is a kind of musical alchemy, pouring rarified ingredients (and more than a drop of our own blood) into a bubbling cocktail of pitches, patterns, modes and memories. We can nudge around neutralities or at extremes, overtly manipulate raw emotions, all the while not really knowing how or why this might make a person cry. But there are some of the tips and tricks that one *can* learn and apply.

At the most fundamental level we use rhythm and dynamics, in other words speed and loudness, to communicate urgency and importance. These musical signals are primal responses. They exist on an iconic level, almost completely independent of culture. There is perhaps no better example of this than the theme from “*Jaws”* (Dir. Steven Spielberg/Composer John Williams, 1975). It is like a musical doppler effect as the shark grows nearer and nearer, faster and louder … Similarly, pitch itself can denote size and gravitas, from ominous and threatening lows to diminutive highs.

The next cornerstones of musical storytelling are tension and release, which can be explored through many means. A movement from mounting dischord to a gentle, resolving harmony. An accelerating rhythm giving way to a sustained statement of arrival.

We can explore theme and variation to great effect in film music. Drawing from *Leitmotif*^3^ techniques in opera, a short theme is used to build a melodic identification with a particular character or situation. Riding this musical familiarity sends useful information to the listener and helps them know what is happening or who we are talking about, on or off screen. We can sense Darth Vader approaching, even if we are not one with The Force, because we hear his tune. We can then create variations on the theme to suit the mood and situation, shifting into major or minor tonalities, accelerating or stretching out its content.

One of the most beautiful examples of this use of themes is in Nino Rota's score for “*The Godfather”* (Francis Ford Coppola, 1972). He introduces a new theme when we meet the young Michael Corleone. It feels innocent and somewhat wistful. The theme develops in intensity and prominence as he grows in power, until the bold glory of Michael's fully-formed theme signals that he has arrived to take his place at the helm of the family.

We can use an unexpected musical shift, a lack of resolution, to surprise and enliven. It could be a breach in musical form or timing, or both together.

We can exploit the timbral variety of the orchestra to great effect. The choices that we make in orchestration can reflect certain emotional traits that these timbral differences offer: the melancholia of the cello, the innocent sadness of the oboe, the mystery of the flute, the boldness of the trumpet, or the transparent beauty of the harp.

We can go deeper and explore timbral differences within the instrument itself, in the form of tessitura. A choice to use the higher range of the cello rather than the lower range of the viola, for example, will propel a slightly different emotional quality. It reaches a little more.

The use of tremolo in sustained string lines creates or reflects tension. Why? Perhaps because human perceptions are geared toward recognizing change—and tremolo is continuous instability, continuous change. So it gives *us* a sense of instability and suspense.

We can present incongruities between picture and music to create a sense of something wrong, something surreal, where meaning is called into question. The opening battle scene of Ridley Scott's film “*Gladiator”* (2000) imposes a gentle, longing orchestral movement on the horror of bloody war as the armies of the Hun are massacred.

Similarly in comedy—whose very foundation is incongruity—you will often see comedic action scored as a serious undertaking. From the perspective of the film's protagonist, these are serious matters. To us it becomes ridiculous.

An oft-used way to impart a sense of continual forward motion is shifting the musical key up in intervals of a third. The musical underpinning is that this modulation “destroys the root” (the root is the note upon which the original chord is built). For some reason the brain jumps to this new place and no longer searches for resolution back to the original key.

These ideas are just some of the many long-used compositional and orchestration vehicles in the western tradition that we can adapt to help tell a story. And they work quite universally. Somewhat surprisingly, emotional cues in music from very diverse cultures can readily be picked up by those without any previous exposure to that kind of music (Balkwill and Thompson, 1999).

## What is music for?

So what exactly is music for? Here are some ideas that interweave:

### Biology

Throughout evolution from the most primitive animal utterings, musical sounds have served our basic biology—attracting and securing a mate, deterring a predator or warning of danger. In more complex species they are a catalyst for bonding and social cohesion (2008). They can both affirm and set aside an animal's individuality. In humans it goes further, expressing shared tastes, moods and a deeper identification with each other and for oneself (Shepherd and Wicke, 1997). Yet at heart, music is an act of interplay. When looking at brain imaging studies (Levitin, 2006), it is not just the composer or improvisor's brain that demonstrates wide activity. The brain of a listener lights up like a Christmas tree, brimming with sympathetic and covert responses, digging into its own understandings and memories and experiences. Indeed as composers, we are not painting a full and complete picture for our listeners of what to feel, we are really just opening a window and pointing in a certain direction. Listeners find their own way there and maybe go way beyond, or somewhere else entirely. That is perhaps what makes music so exciting: we are all *participants*, we are co-creators of all of these musical emotional experiences. We are exploring a shared empathy.

### Abstraction

To be human is to be capable of abstract thinking, of conceptualization, conscious and unconscious. We imagine traveling to the moon on an abstract plane, then we bring it into being. This capacity is both an outgrowth and a tool of our evolution. As defined as music can try to be, it is still fundamentally abstract. More than allowing us to speak in-between-the-lines, perhaps music is representing our capacity for abstract imagining of an environmental condition. Maybe it is echoing environmental cues that have been with us throughout our evolution:

- The throb of low drums remembers the pounding stampede of a herd, the rumble of thunder, a shaking earthquake.
- The oboe or cello is the mournful crying of a grown adult.
- The violin, the cries or playfulness of a child.
- The breathiness of a wooden flute, recalls the whispering of a loved one in your ear.
- The gentle shaking of percussion, remembers the wind through leaves.

### Influence and control

We can also control our emotional environment, much as we control room temperature. We can calm or stimulate ourselves, work a crowd into a dancing frenzy or sing a lullaby to send a child to sleep. One of the more stark examples of musical control is being explored in bus stations and public places where crime tends to hover. Classical music is piped through the speakers in an effort to diminish violent behavior and keep unruly types at bay (Midgette, 2012).

### Transportation

To me, music is an open place, a portal. It is a transportation system to and from our subconsciouses, our instincts and our shared culture. This ancient and amazing technology, still developing from its beginnings of primitively produced sound to birdsong, and all the way to symphonies and salsa. It is a technology that has the power to transport us instantly to a time past or future, yet not in a passive way. We are immersed and we can relive an experience in vivid depth and feeling: the song that played when we first fell in love, or that fueled the wild antics of a summer vacation. Music can transport us to a place or geography—the twang of a sitar, a mountain yodel or an African polyrhythm—each encoding a cultural longitude and latitude. It can transport us to a human emotion, on a journey from sadness to joy. Because after all, “emotion” *means* to move, to move on. And it can transport us to somewhere beyond the human experience, beyond words, beyond thought, beyond our furthest imaginings—to transcendence.

### Conflict of interest statement

The author declares that the research was conducted in the absence of any commercial or financial relationships that could be construed as a potential conflict of interest.

## Footnotes

^1^*Nasrabadi in Attar: Tadhkirat, 793.*

^2^*www.youtube.com/watch?v=2T5_0AGdFic (2006) This recut of the Disney classic “Mary Poppins” was made by Chris Rule with assistance by Nick Eckert. It contains the musical piece “A Violent Attack” composed by Caine Davidson for the film “An American Haunting,” “Stay Awake” written by Richard and Robert Sherman for Disney's “Mary Poppins,” and stock sounds from iMovie.*

^3^*A **leitmotif** /,latmoƱ'ti:f/ is a “short, Constantly Recurring Musical Phrase Associated with a particular Person, Place, or Idea. Richard Wagner is the Earliest Composer Most Specifically Associated with the Concept of Leitmotif.”*
